# Reply to Ravnskov, U. Is High Cholesterol Deleterious? An Alternative Point of View. Comment on “Burén et al. A Ketogenic Low-Carbohydrate High-Fat Diet Increases LDL Cholesterol in Healthy, Young, Normal-Weight Women: A Randomized Controlled Feeding Trial. *Nutrients* 2021, *13*, 814”

**DOI:** 10.3390/nu13072127

**Published:** 2021-06-22

**Authors:** Jonas Burén, Madelene Ericsson, Nágila Raquel Teixeira Damasceno, Anna Sjödin

**Affiliations:** 1 Department of Food, Nutrition and Culinary Science, Umeå University, 90187 Umeå, Sweden; anna.sjodin@umu.se; 2 Department of Public Health and Clinical Medicine, Medicine, Umeå University, 90187 Umeå, Sweden; 3 Department of Medical Biosciences, Physiological Chemistry, Umeå University, 90187 Umeå, Sweden; madelene.ericsson@umu.se; 4 Umeå Centre for Molecular Medicine, Umeå University, 90187 Umeå, Sweden; 5 Department of Nutrition, School of Public Health, University of Sao Paulo, Sao Paulo 05508-060, Brazil; nagila@usp.br

We thank Ravnskov [1] for his interest in our recent publication [2]. In our study, low-density lipoprotein cholesterol (LDL-C) and Apolipoprotein B-100 (ApoB) almost doubled in young, healthy women when they followed the ketogenic low-carbohydrate high-fat (LCHF) diet. According to the European Society of Cardiology (ESC) and the European Atherosclerosis Society (EAS) Guidelines for the management of dyslipidaemias [3] “…there is no longer an ‘LDL-C hypothesis’, but established facts that increased LDL-C values are causally related to atherosclerotic cardiovascular disease, and that lowering LDL particles and other ApoB-containing lipoproteins as much as possible reduces cardiovascular events.” Therefore, we think it is reasonable to conclude that the alterations in blood lipids reported in our study should be a cause for concern in young women following this kind of LCHF diet.
